# Regulation of hematopoietic stem cells differentiation, self-renewal, and quiescence through the mTOR signaling pathway

**DOI:** 10.3389/fcell.2023.1186850

**Published:** 2023-05-09

**Authors:** Bai Ling, Yunyang Xu, Siyuan Qian, Ze Xiang, Shihai Xuan, Jian Wu

**Affiliations:** ^1^ Department of Pharmacy, The Yancheng Clinical College of Xuzhou Medical University, The First People’s Hospital of Yancheng, Yancheng, Jiangsu, China; ^2^ Zhejiang University School of Medicine, Hangzhou, Zhejiang, China; ^3^ The Second School of Clinical Medicine, Wenzhou Medical University, Wenzhou, China; ^4^ Department of Laboratory Medicine, The People’s Hospital of Dongtai City, Dongtai, China; ^5^ Department of Clinical Laboratory, The Affiliated Suzhou Hospital of Nanjing Medical University, Suzhou Municipal Hospital, Gusu School, Nanjing Medical University, Suzhou, Jiangsu, China

**Keywords:** hematopoietic stem cells (HSCS), mTOR, differentiation, self-renewal, quiescence

## Abstract

Hematopoietic stem cells (HSCs) are important for the hematopoietic system because they can self-renew to increase their number and differentiate into all the blood cells. At a steady state, most of the HSCs remain in quiescence to preserve their capacities and protect themselves from damage and exhaustive stress. However, when there are some emergencies, HSCs are activated to start their self-renewal and differentiation. The mTOR signaling pathway has been shown as an important signaling pathway that can regulate the differentiation, self-renewal, and quiescence of HSCs, and many types of molecules can regulate HSCs’ these three potentials by influencing the mTOR signaling pathway. Here we review how mTOR signaling pathway regulates HSCs three potentials, and introduce some molecules that can work as the regulator of HSCs’ these potentials through the mTOR signaling. Finally, we outline the clinical significance of studying the regulation of HSCs three potentials through the mTOR signaling pathway and make some predictions.

## Introduction

Hematopoietic stem cells (HSCs) exist in hematopoietic tissues and play an important role in hematopoiesis. The HSC pool is divided into two different subpopulations according to their long-term reconstituting capacity: long-term HSCs (LT-HSCs) and short-term HSCs (ST-HSCs), which can later differentiate into multipotent progenitors and finally differentiate into lymphoid or myeloid cells with various functions (Morrison and Weissman, 1994; Buitenhuis et al., 2008).

Most HSCs maintain quiescence *in vivo*, which is an important mechanism for maintaining the number of HSCs and hematopoiesis balance. Moreover, HSCs remain in quiescence at a steady state to preserve their self-renewal potential and protect themselves against genetic damage and exhaustive stress to ensure their longevity (Nakamura-Ishizu et al., 2014; Calvi and Link, 2015; Chen Z. et al., 2021). Under stress conditions such as tissue injury and inflammation, HSCs can be activated and enter the cell cycle, starting their self-renewal and differentiation to respond to these emergencies (Eaves, 2015; Dzierzak and Bigas, 2018). In the self-renewal process, HSCs can generate progeny cells that are identical to themselves to maintain pluripotency and increase their number at the same time. For example, if HSCs are transplanted into a patient to treat some diseases, LT-HSCs can self-renew to rehab the impaired blood system after transplantation, which is crucial for normal operation of the blood system and recovery from some serious blood and autoimmune diseases (Chabannon et al., 2018). Moreover, HSCs can differentiate into almost any blood cell under certain conditions, most of which are immune cells. It is important for the immune system to maintain its function through the continuous generation of immune cells from HSCs. In a word, HSCs differentiation, self-renewal, and quiescence are all very indispensable for HSCs, they are tightly associated with hematopoiesis balance and even life-long blood production. If something goes wrong with these processes, unfortunately, not only the blood system function is affected, some serious diseases, such as hematopoietic failure or malignancies may therefore happen as well (Reya et al., 2001; Rossi et al., 2008; Wang et al., 2015b; Xiong et al., 2019; Wu et al., 2020).

The mammalian target of rapamycin (mTOR) is a serine/threonine kinase, which can sense a variety of signals, including environmental and intracellular signals from nutrients, growth factors, and so on. Then, mTOR works as a regulatory center, integrating these signals and starting to regulate various important vital movements (Ning et al., 2022; Wolfe et al., 2023). Due to its powerful regulatory function, the mTOR signaling pathway plays an important role in regulating cell growth, proliferation, metabolism, and so on (Saxton and Sabatini, 2017). mTOR can compose two protein complexes, mTOR complex 1 (mTORC1) and mTOR complex 2 (mTORC2). mTORC1 has mTOR, Raptor, PRAS40, DEPTOR, and mLST8. It is sensitive to rapamycin and regulates important vital movements such as mRNA translation, cell growth, and protein synthesis (Inoki et al., 2002; Wullschleger et al., 2006; Saxton and Sabatini, 2017). mTORC2 also has mTOR, mLST8, and DEPTOR, which are contained in mTORC1. What is different from mTORC1 is that mTORC2 contains Rictor, which makes mTORC2 insensitive to rapamycin. Besides, mTORC2 also includes the regulatory subunits mSin1 and Protor1/2. and it is correlated with gluconeogenesis, cytoskeleton organization, and cell survival (Wullschleger et al., 2006; Hagiwara et al., 2012; Saxton and Sabatini, 2017). The mTOR signaling pathway plays an important role in many physiological processes, and abnormalities in it are implicated in the pathogenesis of many diseases, including cancer (Cai et al., 2014). Studies have found that aberrant regulation of mTOR is a hallmark of many cancers and mTOR can be used as a therapeutic target for cancer (Din et al., 2012; Lastwika et al., 2016; Hua et al., 2019).

Several studies have already revealed that the mTOR signaling pathway can regulate HSCs’ differentiation, self-renewal, and quiescence potentials. Meanwhile, a variety of molecules have already been found to regulate these three potentials by affecting the mTOR signaling pathway. These studies may provide many new approaches to regulate HSCs’ activities and have significance in clinical treatment. In this review, we will discuss how the mTOR signaling pathway functions to regulate these three potentials and some molecules that can regulate these potentials by influencing the mTOR signaling pathway.

### Mechanism of the mTOR signaling pathway regulating HSCs

mTOR signaling pathway is a very essential signaling pathway in the cells. It has been demonstrated that mTOR signaling pathway plays an important role in hematopoiesis and mTOR protein levels vary considerably during hematopoiesis (Hoshii et al., 2014; Spevak et al., 2020).

**FIGURE 1:**
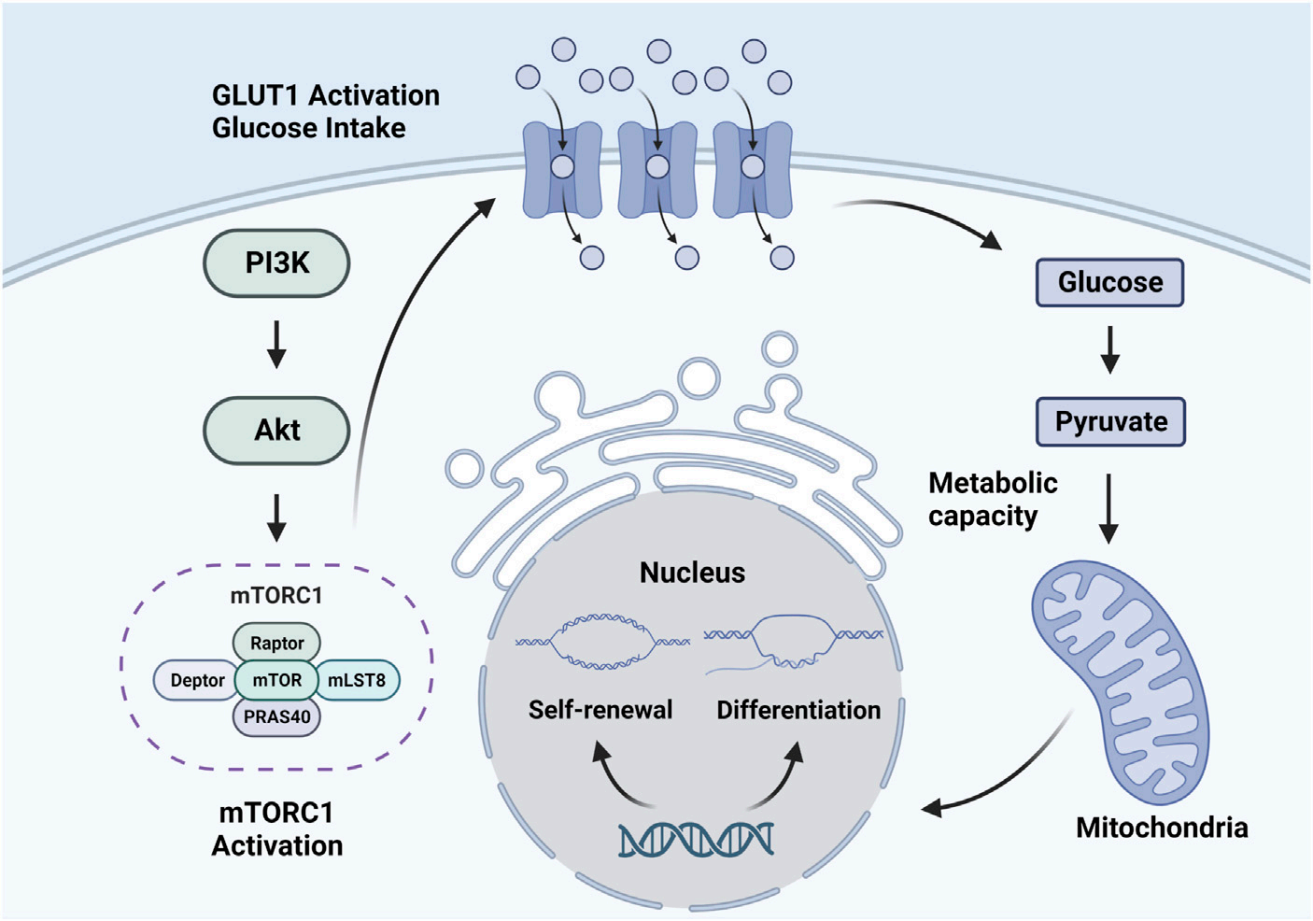
Mechanism of the mTOR signaling pathway regulating HSCs.

The mechanism by which the mTOR signaling pathway regulates the fate of HSCs is not well understood, but some existing studies have provided some evidence (Fernandes et al., 2021). In fact, glucose transporter (GLUT)1 expression is associated with mTOR activation in hematopoietic and non-hematopoietic cells, which is the possible mechanism (Wu et al., 2018; Sharif et al., 2019). As the results of a study by Sarrazy et al. (2016), bone marrow production was impaired in GLUT1 knockout models, indicating the vital role of GLUT1 in the mechanism. High expression level of GLUT1 causes more glucose to enter the HSC. Then, high glucose levels promote HSC metabolism and induce HSCs to quit quiescence and enter self-renewal and differentiation. High glucose levels in HSCs will also lead to O-linked β-N-acetyl glucosamine protein modifications and have regulating effects on HSCs (Saki et al., 2013). What’s more, glucose in HSCs can directly regulate gene expression and influence the function of cyclins, thus regulating the quiescence, self-renewal and differentiation of HSCs (Albert et al., 2019) (Figure 1).

### Quiescence and self-renewal of HSCs through the mTOR signaling pathway

In steady state, the majority of HSCs stay in a quiescent cell cycle state. Studies have shown that less than 5% of HSCs are in the S/G2/M phases of the cell cycle, 20% are in the G1 phase, and more than 70% of HSCs are in the G0 phase (Bradford et al., 1997; Cheshier et al., 1999). This status is called HSC quiescence. For example, in the bone marrow, HSCs proliferate the first 3 weeks after birth and after that they stay in quiescence for a long time (Bowie et al., 2006). These quiescent HSCs divide at a very slow rate and show comparatively smaller cell size, lower transcriptional activity, and metabolic activity. Furthermore, they exhibit deficient RNA content and synthesize protein at an extremely slow rate (Wilson et al., 2008; Foudi et al., 2009; Wilson et al., 2009; Buszczak et al., 2014; Signer et al., 2014; Llorens-Bobadilla et al., 2015; Zismanov et al., 2016; Xiang et al., 2022). Quiescence is of great significance as a basic property of HSCs (Pietras et al., 2011). In detail, quiescence is supposed to prevent functional exhaustion and cell damage, and it can protect HSCs from malignant transformation and malfunction (Calvi and Link, 2015) so that HSCs in quiescence can be preserved for a long time and the number of HSCs can be maintained. In other words, maintain of quiescence is pivotal for HSCs to preserve their function and number.

The self-renewal of HSCs is the ability to produce identical daughter HSCs without differentiation (Seita and Weissman, 2010). HSC is defined by its capacity to continuously produce all types of blood cells. In this definition, HSCs continuous function just reflects their self-renewal feature (Weissman, 2000). In homeostatic conditions, HSCs maintain the potential for long-term self-renewal and the capacity for subsequent reconstitution. However, in some emergencies, severe hematopoietic stresses make HSCs lose this potential (Lee et al., 2018). The self-renewal potential is indispensable for HSCs because the organism needs self-renewal to retain an adequate pool of HSCs, which is important for the balance of hemopoietic function and the normal operation of the hemopoietic system. Furthermore, it should be mentioned that HSC quiescence is important in preserving HSCs self-renewal potential of HSCs (Matsumoto et al., 2011; Kobayashi and Suda, 2012; Qing et al., 2014).

The two isolated populations of HSCs, LT-HSCs and ST-HSCs, have different self-renewal capacities. LT-HSCs have higher self-renewal capacity than ST-HSCs (Haug et al., 2008; Wilson et al., 2008). Self-renewal capacity is also linked to age. Aged HSCs were shown to show reduced self-renewal and regenerative capacities (Bernitz et al., 2016). Regarding HSC self-renewal, the two protein complexes mTORC1 and mTORC2 give play to diverse regulatory roles. mTORC1 works as a down-regulator of HSC self-renewal. The deficiency of the mTORC1 activator Rheb1 leads to expansion *in vitro* and *in vivo*, indicating that the activation of mTORC1 inhibits HSC self-renewal, as does its inhibition of HSC quiescence (Wang et al., 2019). However, mTORC2 doesn’t play an inhibitory role in HSC self-renewal regulation. The deletion of Rictor, the essential component of mTORC2, has unobvious effects on HSC maintenance and self-renewal (Kalaitzidis et al., 2012).

Previous research has clarified that some cell cycle regulators, transcription factors, epigenetic molecules, niche factors, and even physical factors all can regulate HSC switching between quiescence and self-renewal, thus maintaining the balance of hematopoietic (Göttgens, 2015; O'Reilly et al., 2021). Furthermore, a series of kinase-related signaling pathways have been found to regulate quiescence and self-renewal in HSCs synergistically, among which the mTOR signaling pathway is indispensable. It has already been revealed that activation of mTOR, or its upstream protein kinase B (Akt), obviously induces quiescent HSCs to exit quiescence and enter self-renewal progress (Chen et al., 2008; Juntilla et al., 2010; Kharas et al., 2010; Hemmati et al., 2019).

Here are some molecules that have been shown to regulate HSC switching between quiescence and self-renewal by influencing the mTOR signaling pathway (Table 1). The nuclear gene DEK has been found in recent research to have something to do with HSC quiescence. Chen *et al.* showed that DEK protein upregulated the expression of genes associated with cell cycling but inhibited the expression of genes associated with quiescence maintenance in HSCs. It is partly because DEK deficiency activates mTOR signaling, and then HSCs are induced to exit quiescence and enter cell cycles (Chen Z. et al., 2021). Another study showed that Sel1L/Hrd1 endoplasmic reticulum-associated degradation (ERAD) complex is more active in quiescent HSCs, and the expression of Sel1L/Hrd1 genes is reduced when HSCs exit quiescence. Mechanically, Ras homolog enriched in brain (Rheb), a small GTPase, may function as a substrate of ERAD, which links Sel1L/Hrd1 ERAD to mTOR activation (Liu et al., 2020). Rheb accumulates in activated HSCs and it is known to directly activate mTORC1 (Saucedo et al., 2003; Liang et al., 2020). When Liu *et al.* eliminated Sel1L, the ERAD deficiency leads to the accumulation of Rheb proteins, thus activating mTORC1 in HSCs. Therefore, HSCs are induced to exit quiescence (Liu et al., 2020). In this way, Sel1L/Hrd1 ERAD functions as a positive regulator that keep HSCs in quiescence. The tuberous sclerosis complex (TSC) can inhibit the target of rapamycin, and the genetic lesions of two genes, TSC1 and TSC2, are the cause (Consortium, 1993; Slegtenhorst et al., 1997). Among them, the TSC1 protein can function as a negative regulator of mTORC1 (Inoki et al., 2002; Potter et al., 2002). Previous research found that TSC1 deletion led to mTOR activation, which could show that TSC1 preserves the quiescence of HSCs by inhibiting the activation of mTOR (Saucedo et al., 2003).

**TABLE 1:** Molecules that regulate HSC quiescence and self-renewal through mTOR signaling pathway.

Molecules	Effects on mTOR	Regulation of HSC quiescence and self-renewal	References
DEK protein	Inhibiting mTOR signaling	Up-regulating quiescence maintenance-associated genes to keep HSCs in quiescence	Chen et al. (2021b)
Sel1L/Hrd1 ERAD complex	Inhibiting the accumulation of Rheb proteins to inhibit the activation of mTORC1	Keeping HSCs in quiescence	Liu et al. (2020)
TSC1 protein	Working as a negative regulator of mTORC1	Preserving HSC quiescence	Saucedo et al. (2003)
Fbxw7	Inducing the mTOR protein degradation	Keeping HSCs in quiescence	Iriuchishima et al. (2011)
Itpkb	Inhibiting Akt/mTORC1 activation in HSC	Promoting HSC quiescence	Siegemund et al. (2015)
Srebf1c	Inhibiting hyper-activation of mTORC1	Keeping HSCs in quiescence	Schmidt (2013)
FLCN	Inhibiting hyper-activation of mTORC1	Keeping HSCs in quiescence	Baba et al. (2016)
PTEN	Working as a down-regulator mTOR	Keeping HSCs in quiescence	Yilmaz et al. (2006)
PML	Working as a down-regulator mTOR	Keeping HSCs in quiescence	Ito et al. (2008)
miR-1246	Causing the suppression of the mTOR subunit Raptor and inhibiting protein synthesis	Keeping HSCs in quiescence	Abdelhamed et al. (2019)
Lysosomes	Degradation of lysosomal cargo reducing ROS levels	Inducing HSCs to exit quiescence and enter self-renewal	Liang et al. (2020)
GSK-3β	Functioning downstream of PTEN	Maintaining HSC self-renewal	Huang et al. (2009)
ACA	Influencing the PI3K/Akt/mTOR/PTEN pathway	Up-regulating the expression of important genes to induce HSC self-renewal	Becker-Kojić et al. (2013)
Myh9	Maintaining mTOR related pathway gene sets	Preserving HSC self-renewal capacity	An et al. (2022)
Dlk1-Gtl2 locus	Suppressing the entire PI3K-mTOR pathway	Maintaining HSC self-renewal capacity	Qian et al. (2016)
PRMT5	Inhibiting the activation of mTOR	Preserving HSC self-renewal capacity	Tan et al. (2019)

Some other proteins can also maintain HSCs quiescence through the mTOR signaling pathway. F-box and WD-40 domain protein 7 (Fbxw7), the component of the F-box protein of a stem cell factor–type ubiquitin ligase, can also regulate the quiescence of HSCs. The deletion of Fbxw7 in adult HSCs results in loss of quiescence in HSCs (Iriuchishima et al., 2011). mTOR is one of the substrates of Fbxw7 (Mao et al., 2008). Forced expression of Fbxw7α, the main Fbxw7 isoform in HSCs, induces the degradation of the mTOR protein, thus keeping HSCs in quiescence (Iriuchishima et al., 2011). Itpkb is also found to function as an important regulator of HSC homeostasis which can ensure HSCs’ quiescence (Siegemund et al., 2015). Proto-oncogenic class I phosphoinositide-3-kinases (PI3K) and their effectors such as Akt, mTORC1 and mTORC2 are important regulators of HSCs (Saucedo et al., 2003; Gan et al., 2008; Ito et al., 2012; Liang et al., 2020; Liu et al., 2020). Siegemund *et al.* showed that Itpkb has an important role in regulating HSCs homeostasis. The mechanism is that Itpkb inhibits cytokine-induced PI3K signaling, thus inhibiting Akt/mTORC1 activation in HSCs. Therefore, Itpkb can act as a positive regulator that promotes HSC quiescence (Siegemund et al., 2015). Sterol regulatory element binding factor-1c (Srebf1c) works as a significant metabolic regulator in both humans and mice (Wang et al., 2015a). Studies found that Srebf1c also plays an important role in HSC quiescence. The deficiency of Srebf1c causes the hyperactivation of mTORC1, which promotes HSCs to quit quiescence and enter cell cycles. Therefore, Srebf1c also helps HSCs remain in quiescence by inhibiting the overactivation of mTORC1 in the mechanism (Schmidt, 2013). Folliculin (FLCN) is a multifunctional protein that can modulate a variety of signaling pathways playing important roles in cells activities like growth, metabolism, proliferation, adhesion, and survival (Schmidt, 2013; Tee and Pause, 2013). The research found that FLCN also functions to maintain the quiescence of adult HSCs (Baba et al., 2016). Mechanically, mTORC1 activation may explain why HSCs quit quiescence and hematopoietic failure occurs with loss of FLCN (Baba et al., 2016).

In addition, many studies have found some other types of negative regulators of mTOR that can help keep HSCs in a quiescent state, such as PTEN (Yilmaz et al., 2006; Zhang et al., 2006) and PML (Ito et al., 2008). The deletion of them leads to the hyperactivation of mTOR, then HSCs are induced to quit quiescence and enter active cell cycling.

There are some special ways to maintain the quiescence of HSCs. Acute myeloid leukemia (AML), a genetically heterogeneous disease, has something to do with the quiescence of HSCs. Extracellular vesicles (EV) comprise many nanosized vesicles that carry protein and nucleic acids (Butler et al., 2018). AML-EV is abundant in micro-RNA (miR). The research found that AML-EV transfer miR-1246 to LT-HSCs to cause the suppression of the mTOR subunit Raptor, thus inhibiting protein synthesis in LT-HSCs. Therefore, AML-EV induces HSC quiescence (Abdelhamed et al., 2019). Recent research also showed that repression of lysosomal activation is essential for the maintenance of HSC quiescence. The slow degradation of lysosomal cargo reduces ROS levels and possibly modulates amino acid efflux and mTOR activation. Hence, inhibiting lysosomal activation can prevent the hyper-activation of mTOR, thus keeping HSCs in quiescence (Liang et al., 2020).

There are also some molecules that can induce HSCs to quit quiescence and start self-renewal by affecting the mTOR signaling pathway (Table 1). Previous research found that glycogen synthase kinase-3 (GSK-3) plays an essential role in regulating the balance between self-renewal and lineage commitment in HSCs. GSK-3 has two subtypes, GSK-3α and GSK-3β. Mechanically, GSK-3βfunctions downstream of PTEN to induce HSCs self-renewal (Huang et al., 2009).

ACA is a novel human GPI-linked surface glycoprotein that was previously isolated from human blood (Kojić and Terness, 2002). A former study indicated ACA is a unique regulator of HSCs self-renewal. Researchers demonstrated that the signaling machinery initiated by the cross-linking of ACA is capable of generating and maintaining human primitive self-renewing cells. The mechanism is that ACA promotes the generation of primitive hematopoietic self-renewing cells by upregulating the expression of important genes known to participate in the control of self-renewal through the PI3K/Akt/mTOR/PTEN pathway (Becker-Kojić et al., 2013).

Myosin heavy chain 9 (MYH9) also has something to do with HSC self-renewal according to a recent study (An et al., 2022). An *et al.* observed that Myh9 deficient HSCs were functionally hampered to develop into mature hematopoietic precursors and severely defective in repopulation capacity. That is, the loss of the gene Myh9 seriously inhibits the self-renewal of HSCs. Therefore, these HSCs cannot maintain long-term hematopoiesis. The result of the Gene Set Enrichment Analysis showed that the gene sets were negatively regulated in Myh9 deficient cells, which may indicate that inhibition of HSC self-renewal caused by Myh9 deficiency is in part due to the impaired mTOR signaling pathway (An et al., 2022).

The Dlk1-Gtl2 locus which contains the largest cluster of miRNAs also participates in the regulation of HSC self-renewal. Some researchers have linked the Dlk1-Gtl2 locus to energy metabolism (Charalambous et al., 2012; Labialle et al., 2014). Loss of imprinting at the Dlk1-Gtl2 locus induced hyperactivation of the PI3K-mTOR Pathway, thus affecting the self-renewal capacity. The study of Qian et al. (2016) further revealed that the miRNA cluster in the Gtl2 locus suppresses the entire PI3K-mTOR pathway and inhibits mitochondrial activity to maintain HSC self-renewal capacity. Therefore Dlk1-Gtl2 locus plays a positive regulatory role in HSC self-renewal.

Moreover, Tan *et al.* found that the Protein arginine methyltransferase 5 (PRMT5) is associated with HSCs self-renewal. Researchers found that reduction in PRMT5 activity increases mTOR signaling and protein synthesis, which constitutes a major branch of the proteostasis network (Klaips et al., 2018). In the study, HSC loss was observed upon depletion of PRMT5 activity, which was due to activation of mTOR caused by the deficiency of PRMT5 activity. Thus, PRMT5 is an up-regulator of HSC self-renewal which functions by inhibiting mTOR hyper-activation. However, how reduced PRMT5 activity specifically causes mTOR activation is still unclear and requires further studies (Tan et al., 2019).

In summary, a wide variety of molecules can regulate HSC quiescence and self-renewal by influencing the mTOR signaling pathway. In terms of mechanism, most regulators preserve HSC quiescence or induce HSC self-renewal by the regulation of mTORC1 activation, further highlighting the significance of mTORC1 in the switch between HSC quiescence and renewal.

### Differentiation of HSCs through the mTOR signaling pathway

In the above, it is mentioned that HSC is defined by its ability to continuously give rise to all types of blood cells. The capacity to give rise to all types of blood cells is the differentiation potential. It indicates that HSCs have the multipotency to differentiate into all functional blood cells, which is completely different from the self-renewal capacity that produces identical daughter HSCs (Seita and Weissman, 2010). This differentiation potential is also very indispensable for HSCs because the blood cells derived from HSCs are essential in life activities such as blood circulation and immune regulation.

The blood system reflects the balance of two essential abilities of HSCs, self-renewal and differentiation. Although mature blood cells are generated at a high speed of more than one million cells per second in adult humans (Ogawa, 1993), most of the HSCs from which they are derived remain in a quiescent state and reside in the G0 phase of the cell cycle under homeostatic conditions, as mentioned above. In other words, only a small part of HSCs participates in the generation of blood cells. Therefore, the balance of HSC self-renewal and differentiation is essential for the organism to retain a sufficient pool of HSCs and consistently meet its own enormous demand for continuous replenishment of short-lived mature blood cells at the same time (Seita and Weissman, 2010). Furthermore, it has been shown that in humans and mice, hematopoietic differentiation programs appear to inhibit the self-renewal (van Galen et al., 2014).

As an important signaling pathway, the mTOR signaling pathway also influences HSC differentiation. Research has found that mTOR deficiency caused a reduction in blood lineage production, indicating that mTOR may play an important upregulation role in HSCs differentiation (Guo et al., 2013). Like the regulation of HSC self-renewal, mTORC1 and mTORC2 also play different roles in the regulation of HSC differentiation (Kalaitzidis et al., 2012). This was demonstrated by the selective knockout of Raptor and Rictor. These experiments demonstrated that Raptor plays an important role in HSC differentiation; conversely, Rictor has little impact on it. Therefore, it revealed the significance of mTORC1 in regulating HSC differentiation (Kalaitzidis et al., 2012; Magee et al., 2012). What’s more, some researchers found that mTORC1 activation brings about HSC differentiation *in vitro* and *in vivo*, and this effect can be inhibited by rapamycin (Notario et al., 2018; Tan et al., 2019). This further proved that mTORC1 is essential in the differentiation potential, which functions as a positive regulator. According to the previously stated, mTORC1 can activate GLUT1, thus causing high glucose levels in HSCs. Then, signal transducer and activator of transcription (STAT)5, which is a regulator of HSC differentiation, can be activated by O-linked β-N-acetyl glucosamine (Freund et al., 2017). In this way, the differentiation of HSCs can also be upregulated.

There are some molecules that can regulate HSC differentiation by infecting the mTOR signaling pathway as well (Table 2). The toll-like receptor 7 (TLR7), which is important in immune recognition, can induce PI3K/mTOR signaling. During infection, it is important for myelopoiesis. It is very likely that TLR7 induces the PI3K/mTOR signaling, thus promoting HSCs myeloid differentiation (Buechler et al., 2016; Fernandes et al., 2021). Another study found that when not infected, TLR7 plays a similar role. In mice, TLR7 caused massive myeloid expansion, which further revealed TLR7’s regulatory function of HSC differentiation (Buechler et al., 2013). In this way, TLR7 functions as an up-regulator of HSC differentiation through the function of the mTOR signaling pathway. Another toll-like receptor, TLR2, is found relative to HSCs myeloid differentiation in a recent study as well. The study has revealed that the TLR2 ligand induces direct myeloid differentiation of HSCs and hematopoietic progenitor cells (HPCs). Mechanically, in response to TLR2 signaling, mTOR induces the differentiation of HSCs and HPCs by directly activating transcription factors involved in myeloid differentiation (Bono et al., 2022).

**TABLE 2:** Molecules that regulate HSC differentiation mTOR signaling pathway.

Molecules	Effects on mTOR	Regulation of HSC differentiation	References
TLR7	Inducing the PI3K/mTOR signaling	promoting HSC myeloid differentiation	Buechler et al. (2016)
TLR2	Inducing mTOR signaling	Activating transcription factors to induce HSC myeloid differentiation	Bono et al. (2022)
Rheb1	Directly activating mTORC1	Working as a positive regulator of HSC differentiation	Wang et al. (2019)
MkMPs	Targeting PTEN to activate mTOR signaling	Inducing HSC differentiation	Kao et al. (2022)

In another research, Wang *et al.* knocked out Rheb1 and found myeloid differentiation was impaired (Wang et al., 2019). As mentioned above, Rheb1 can directly activate mTORC1, so Rheb1 can also work as a positive regulator of HSC differentiation. Furthermore, the ROS level also correlates with HSC differentiation through the mTOR signaling pathway. It was found when in ROS high levels, such as those found in the aging bone marrow microenvironment, HSCs differentiation was induced (Ludin et al., 2014). In the above, we introduced that mTOR can regulate the ROS level, but the regulatory function seems to be bidirectional, that is to say, ROS can as well regulate mTOR in some way (Itoh et al., 2019).

Moreover, Megakaryocytes release sub-micron size microparticles (MkMPs) are also shown to regulate HSCs differentiation in recent research. The study results suggested that MkMPs activate mTOR signaling in HSCs and HPCs to induce Mk differentiation (Kao et al., 2022). The small RNAs in MkMPs play important roles in this regulation process, especially two miRs, miR-486-5p and miR-22-3p which are highly enriched in MkMPs. It is probable that miR-486-5p from MkMPs directly targets PTEN, the negative regulator of PI3K/Akt signaling in the development (Zhang et al., 2006; Polak and Buitenhuis, 2012), and activates PI3K/Akt/mTOR signaling in HSCs. Furthermore, the data suggest that miR-22-3p plays an important role in cell proliferation and Mk maturation (Kao et al., 2022). Therefore, MkMPs regulate the differentiation in these ways.

As we can see, the regulation mechanism of HSCs’ differentiation is different from that of their quiescence and self-renewal, almost the opposite. Therefore, it is reasonable that some researchers found hematopoietic differentiation programs seem to inhibit HSCs self-renewal.

### Significance in the clinical treatment

As mentioned above, mTOR exactly plays an indispensable role in the regulation of the quiescence, self-renewal, and differentiation potentials of HSCs, and researchers have already found many molecules that can regulate HSCs these three potentials of HSCs through the mTOR signaling pathway. The significance of these studies involves various aspects (Chen Z.-R. et al., 2021; Li and Chen, 2022). We think that one of the most important aspects is related to clinical treatment. For example, Hematopoietic stem cell transplantation (HSCT) is an important clinical treatment method for a variety of refractory malignant hematopoietic diseases, such as myelodysplastic syndrome, T cell lymphomas, multiple myeloma, and chronic myelomonocytic leukemia (Singh and McGuirk, 2016; De Witte et al., 2017; Schmitz et al., 2018; Gonsalves et al., 2019; Cui et al., 2021). However, the clinical success rate of HSCT isn’t that high, because the successful application of HSCT is frequently inhibited by relapse and graft-versus-host disease (GvHD) (Bhatia et al., 2017; Ghimire et al., 2017). Many methods are tested to inhibit the influence of GvHD to Improve the success rate of HSCT. Inspired by some research, mTOR inhibitors have also been introduced to this field (Abouelnasr et al., 2013; Ziakas et al., 2014). The pathogenesis of GvHD is partly attributed to the activation of donor T cells (Ghimire et al., 2017), so a recent study aims to target the mTOR signaling pathway in T cells to inhibit their activation, thus improving the success rate of HSCT (Zhou et al., 2022). Try to think differently, if we can use the above ways to target the mTOR signaling pathway in HSCs to reduce the differentiation of them, or reduce their differentiation into T cells specificity, it may also make the successful application of HSCT easier to achieve.

Leukemia is a kind of malignant clonal disease of HSCs, and some of the leukemia stem cells (LSCs) directly originate from malignant transformation of normal HSCs. More and more studies have revealed that the imbalance of PI3K/Akt/mTOR signaling leads to leukemogenesis, and increased activity of mTORC1 and mTORC2 has been shown to play a critical role in the regulation of the initiation, propagation and relapse of leukemia (Beauchamp and Platanias, 2013; Fang et al., 2017; Evangelisti et al., 2018). Therefore, there is a possibility that we can use the above ways to regulate HSCs potential to prevent the occurrence of leukemia early.

Anemia is defined as a condition in which the body has a decreased number of circulating erythrocytes or red blood cells (Vieth and Lane, 2014). That is to say, it is caused by the deficiency of red blood cells. If we can use some ways to regulate the mTOR signaling pathway in HSCs to induce them to differentiate into red blood cells more, the symptoms of anemia may be lessened.

Of course, the significance of these studies in clinical treatment is not limited to these diseases. Due to the regulatory role of mTOR in the vital activities and metabolism of stem cells, studies on mTOR may significantly promote the development of stem cell therapy. Therefore, further research is still required, not only on mTOR itself, but also on the whole PI3K/Akt/mTOR signaling pathway and associated regulatory molecules.

## Conclusion

In this review, we discuss the function of the mTOR signaling pathway in the three important potentials, quiescence, self-renewal, and differentiation, all of which are significant in the function of the hematopoietic system. Furthermore, we introduced many molecules that can regulate these three potentials by influencing the mTOR signaling pathway. The regulatory function of some of the molecules was found previously, while that of others was studied recently. And with the continuous development of science and technology, more and more molecules may be shown to regulate these potentials through mTOR signaling.

When discussing the mechanism mTOR regulates HSCs these three potentials, it is obvious that mTORC1 plays a more significant role than mTORC2 in regulating every potential. mTORC1 functions as a negative regulator of HSC quiescence and self-renewal, and its activation can induce HSC differentiation. Therefore, it may also explain why HSCs quiescence seems to be favorable to their self-renewal and why there is an interaction of reciprocal inhibition between HSCs self-renewal and differentiation. However, the function of mTORC2 in regulating HSCs these potentials seems to be a lot weaker compared with that of mTORC1 for some studies observed inapparent changes of HSCs these three potentials with the deficiency of mTORC2. However, we think that mTORC2 may also regulate the three potentials in some ways, and further studies are needed to clarify the function of mTORC2 in this regard.

Furthermore, a variety of molecules have been found to regulate these three potentials by influencing the mTOR signaling pathway. Some of their regulatory function was found previously while others are found recently. And many other molecules may be found to have a similar regulatory function in the future. These studies provide new ideas for regulating the three potentials and may be significant in clinical treatment. If the results of these studies can be applied to HSC-related treatment methods, such as HSCT, the success rate of these studies may be promoted. Besides, these studies may be favorable for the treatment of blood-related diseases as well, including leukemia and anemia. Expect the significance in clinical treatment, the significance in many other aspects is waiting to be discovered in further studies.

In our perspective, existing studies mainly focus on mTOR and its upstream signaling molecules, such as Akt and PI3K. While molecules downstream of mTOR draw little attention. Future research perhaps should focus more on the downstream of mTOR to further elucidate the specific mechanisms by which mTOR regulates various cellular life activities and cellular metabolism.
